# Selected papers from the 15th and 16th international conference on Computational Intelligence Methods for Bioinformatics and Biostatistics

**DOI:** 10.1186/s12859-021-04027-5

**Published:** 2021-04-26

**Authors:** Paolo Cazzaniga, Maria Raposo, Daniela Besozzi, Ivan Merelli, Antonino Staiano, Angelo Ciaramella, Riccardo Rizzo, Luca Manzoni

**Affiliations:** 1 Department of Human and Social Sciences, University of Bergamo, Piazzale Sant’Agostino 2, 24129 Bergamo, Italy; 2 Physics Department, Faculty of Science and Technology, Universidade Nova de Lisboa, Campus de Caparica, 2829-516 Caparica, Portugal; 3 Department of Informatics, Systems and Communication, University of Milano-Bicocca, Viale Sarca 336, 20126 Milan, Italy; 4 Institute for Biomedical Technologies, National Research Council, Via Fratelli Cervi, 93, 20090 Segrate, Italy; 5 Department of Science and Technology, University of Naples “Parthenope”, Centro Direzionale, C4 Island, 80143 Naples, Italy; 6 CNR-ICAR, National Research Council of Italy, Via Ugo La Malfa 153, 90146 Palermo, Italy; 7 Department of Mathematics and Geosciences, University of Trieste, Via Valerio 12/1, 34127 Trieste, Italy

## Introduction

This supplement contains seven revised and extended papers selected from CIBB 2018 and CIBB 2019, the 15th and 16th editions of the international conference on Computational Intelligence Methods for Bioinformatics and Biostatistics. CIBB is a venue that embraces researchers with different backgrounds, ranging from mathematics to computer science, from materials science to medicine, and from engineering to biology, all interested in the investigation and application of computational intelligence methods to open problems in bioinformatics, biostatistics, systems biology, synthetic biology, and medical informatics.

CIBB 2018 was held at Faculdade de Ciências e Tecnologia, Universidade NOVA de Lisboa, Caparica, Portugal, from 6th to 8th September, 2018 [1] and was organized and supported by the Center of Physics and Technological Research (CEFITEC), Physics Department, Faculdade de Ciências e Tecnologia, Universidade NOVA de Lisboa, Portugal. The program of this edition was organized with contributions on the main conference scientific area with heterogeneous open problems at the forefront of current research, and in special sessions on specific themes as Computational Methods for Neuroimaging Analysis, Machine Learning in Health Informatics and Biological Systems, Soft Computing Methods for characterizing Diseases from Omics Data, Engineering Bio-Interfaces and Rudimentary Cells as a way to Develop Synthetic Biology, Modelling and Simulation Methods for System Biology and System Medicine, Fast and Efficient Solutions for Computational Intelligence Methods in Bioinformatics, Systems, and Computational Biology, Networking Biostatistics and Bioinformatics, Machine Explanation—Interpretation of Machine Learning Models for Medicine and Bioinformatics.

CIBB 2019 was held at the Department of Human and Social Sciences of the University of Bergamo, Italy, from the 4th to the 6th of September 2019 [2]. The organization of this edition of CIBB was supported by the Department of Informatics, Systems and Communication of the University of Milano-Bicocca, Italy, and by the Institute of Biomedical Technologies of the National Research Council, Italy. Besides the papers focused on computational intelligence methods applied to open problems of bioinformatics and biostatistics, the works submitted to CIBB 2019 dealt with algebraic and computational methods to study RNA behaviour, intelligence methods for molecular characterization and dynamics in translational medicine, modeling and simulation methods for computational biology and systems medicine, and machine learning in healthcare informatics and medical biology. A supplement published in BMC Medical Informatics and Decision Making journal [3] collected three revised and extended papers focused on the latter topic.

CIBB dates back to 2004, and it was organized as a special session of different Italian conferences up to 2007. In particular, CIBB was part of WIRN 2004 in Perugia, WILF 2005 in Crema, FLINS 2006 in Genoa, and WILF 2007 in Camogli. Afterwards, the Steering Committee of CIBB planned the 2008 edition in Vietri (Italy) as an independent conference. From that moment onward, by relying on the increasing support of the community, CIBB has been organized as a conference, first held only in Italian venues—Genoa (2009), Palermo (2010) and Gargnano (2011)—then, starting from 2012, with an international scope. CIBB 2012 was organized in Houston (TX), then in Nice (France) in 2013, Cambridge (UK) in 2014, Naples (Italy) in 2015, Stirling (UK) in 2016, Cagliari (Italy) in 2017, Lisbon (Portugal) in 2018, and Bergamo (Italy) in 2019.

Thanks to the synergistic effort of the Organizing, Program and Steering Committees, and to the support of sponsors and participants, the success of CIBB conference series is still growing.

## Data Availability

Not applicable.
